# Corneal transplantation for Fuchs´ endothelial dystrophy: A comparison of three surgical techniques concerning 10 year graft survival and visual function

**DOI:** 10.1371/journal.pone.0203993

**Published:** 2018-10-05

**Authors:** Jeroen van Rooij, Elsina H. Lucas, Annette J. Geerards, Lies Remeijer, Rene Wubbels

**Affiliations:** Rotterdam Eye Hospital, Rotterdam, The Netherlands; The Chinese University of Hong Kong, HONG KONG

## Abstract

**Objective:**

Comparison of conventional Penetrating Keratoplasty (PKP), posterior mushroom PKP and Descemet's Stripping Automated Endothelial Keratoplasty (DSAEK) regarding overall graft survival of primary corneal transplants for Fuchs´ endothelial dystrophy (FED), best spectacle-corrected visual acuity (BSCVA) and astigmatism.

**Methods:**

Single centre study using prospectively collected data from the national database for follow-up of corneal transplants. Main outcome parameters: 10 years graft survival, astigmatism at 24 months, pre- and post-operative BSCVA.

**Results:**

In total, 721 cases were included: PKP, n = 171; posterior mushroom PKP, n = 91; and DSAEK, n = 459. There was no significant difference in graft survival between PKP, posterior mushroom PKP and the DSAEK technique (log-rank test, P = 0.12). The overall post-operative BSCVA improvement in all treatment groups was significant (paired t-test, P<0.001). Pre-operative BSCVA was better for the DSAEK group (0.68 ± 0.41 logMAR) as compared to the PKP (0.89 ± 0.53) and posterior mushroom PKP group (0.90 ± 0.58); ANOVA, P<0.001.

After 24 months, BSCVA was significantly better for the DSAEK group (0.25 ± 0.26 logMAR) compared to the PKP (0.35 ± 0.29) and posterior mushroom PKP group (0.41 ± 0.42); ANOVA, P<0.001. A significant difference in astigmatism was found (median test, P<0.001) between the DSAEK (1.7 ± 1.1 D), PKP (4.6 ± 2.7 D) and posterior mushroom PKP group (4.5 ± 3.3 D). The significantly lower DSAEK-induced astigmatism was confirmed by vector analysis.

**Conclusion:**

There was no difference in graft survival and BSCVA *improvement* between conventional PKP, inverted mushroom PKP and DSAEK in this study. The significantly lower changes in astigmatism, wound stability and faster visual rehabilitation with DSAEK surgery are favourable aspects of this technique. The benefits of posterior lamellar keratoplasty warrant earlier intervention, which may contribute to preserve better vision for a prolonged period of remaining lifetime.

## Introduction

Fuchs’ endothelial dystrophy (FED) is a degenerative ocular disease characterized by bilateral accelerated endothelial cell loss with focal outgrowths called guttae and thickening of Descemet’s membrane, leading to corneal edema and loss of vision. Being a major indication for corneal transplantations, FED accounts for 26% of the keratoplasties performed in the USA[1].

During the past decades, keratoplasty procedures for endothelial disorders such as FED have evolved gradually from penetrating keratoplasty (PK) through posterior mushroom PK to endothelial keratoplasty (EK) techniques like descemet stripping automated endothelial keratoplasty (DSAEK) and descemet membrane endothelial keratoplasty (DMEK)[2]. In order to diminish postoperative astigmatism and to transplant a larger area of endothelial cells, the posterior mushroom or ‘tophat’ PK configuration was introduced during the last decade of the past century[3]. The EK techniques avoid some disadvantages of PK techniques such as a large surgical wound inducing a larger postoperative astigmatism leading to spectacle- or hard contact lens dependency.

Only few randomised studies with mostly a limited number of patients were undertaken to compare functional postoperative results like postoperative visual acuity (VA) and refractive error after regular penetrating KP, posterior mushroom KP or posterior lamellar techniques[4,5,6,7]. Recently, a (non-randomised) study compared outcomes of penetrating keratoplasty and DSAEK[8].

The purpose of this retrospective study with a structured prospective follow-up was to report graft survival, the differences in postoperative visual acuity and refractive changes for three transplantation techniques (PKP, posterior mushroom PKP and DSAEK) applied to FED eyes operated in the Rotterdam Eye Hospital (REH). The results give insight in the relative value of the three techniques for patients suffering from Fuchs endothelial dystrophy.

## Materials and methods

In this retrospective, single center study, cornea transplant data were collected from the Dutch Organ Transplant Register (NOTR, financially supported by the Dutch Transplant Foundation (NTS). Data for this register are collected prospectively at fixed follow-up visits with measurements performed in accordance with standard procedures, and data entry by either the surgeon (preoperative and perioperative, graft failures) or the optometrist (other follow-up data). This study adhered to the principles of the Declaration of Helsinki and the ICH guideline for Good Clinical Practice, and was approved by the local Institutional Review Board of the Rotterdam Eye Hospital (REH). As all data were analysed anonymously, no written or oral consent was required.

We retrieved data from patients with Fuchs' endothelial dystrophy who received a primary graft in the REH between 2002 and 2013. Three types of transplantation techniques that were used in the REH during that period were compared: penetrating keratoplasty (PKP), posterior mushroom PKP and Descemet stripping automated endothelial keratoplasty (DSAEK). Donor corneas were stored until shortly before transplantation in organ culture medium at 31°C. Four experienced surgeons performed all transplants. Donor corneas for PKP were punched with a Hessburg Barron (HB) trephine with the endothelial side up (donor oversize 0.5 mm). Recipients for PKP procedures were punched with a HB vacuum trephine. For posterior mushroom PKP, the donor cornea was mounted on a Moria artificial anterior chamber using a 7.5 mm HB vacuum trephine penetrating 65% of the donor cornea with subsequent lamellar dissection towards the limbal side of the corneoscleral button. Subsequently, the donor was trephined at 9.0 mm either from the epithelial side on the artificial chamber or from the endothelial side on a HB punch block; the recipient was typically trephined with a HB vacuum trephine at 7.0 mm. DSAEK donor tissue was prepared at the operating theatre with a Moria artificial anterior chamber and a microkeratome aiming at a posterior lamellar thickness of 80–200 μm. As a rule, the DSAEK graft was inserted through a corneoscleral incision of 4.5 mm with the aid of a Busin- or Tan glide with forceps. All nylon sutures were removed within 2 years after surgery and mersilene sutures were removed selectively.

The postoperative immunosuppressive regimen after all penetrating grafts consisted of dexamethasone 0.1% 6 times daily for 1 month followed by QID up to 3 months, TID from 3–6 months and then tapered down to once daily at 12 months. After DSAEK procedures prednisolone acetate 1% was administered 6 times daily during the first postoperative month followed by QID for two additional months and successively tapered to once daily at 12 months. In case of clinically significant steroid induced intra-ocular pressure elevation, fluorometholone was prescribed in all 3 study groups.

Data at baseline, and at 1, 2, 5 and 10 years postoperatively comprised demographic characteristics, best spectacle-corrected visual acuity (BSCVA), subjective refraction (spherical equivalents), corneal topography (Pentacam, Oculus GmbH), adverse events and graft survival. Statistical calculations and the preparation of graphs were performed using Excel (Microsoft Office 2010) or SPSS (IBM-SPSS, version 20). Visual acuity, measured with the Snellen optotype, was converted to logMAR for analysis. Changes of BSCVA and refraction within groups were evaluated with a two-sided paired t-test. For comparisons between groups, a one-way ANOVA was used (with a post-hoc Tukey test) or, for non-parametric analysis, an Independent Samples Median test. Categorical data were inspected with a χ^2^-test. Kaplan-Meier analysis (log-rank test) was performed to compare failure rates of the three transplantation techniques. Astigmatism was determined from the keratometric measurements (k1/k2 and steep meridian). In order to calculate the change of cylinder (by vector analysis), the polar coordinates (i.e. magnitude & angle) were converted to Cartesian coordinates[9].

## Results

Between January 2002 and January 2013, 721 primary corneal transplants for FED were performed in the REH, i.e. PKP, n = 171; posterior mushroom PKP, n = 91; and DSAEK, n = 459. Baseline characteristics are displayed in Table 1.

**Table 1:** Baseline characteristics–on a per eye basis—of gender, age and laterality differentiated by the technique that was used for corneal transplantation.

	Penetrating Keratoplasty (PKP)	Posterior Mushroom PKP	Descemet Stripping Automated Endothelial Keratoplasty (DSAEK)	All
M / F (total)	70 / 101 (171)	42 / 49 (91)	208 / 251 (459)	320 / 401 (721)
age (mean ± SD)	72.3 ± 9.4	73.6 ± 8.0	71.0 ± 9.2	71.6 ± 9.1
OD / OS (total)	96 / 75 (171)	46 / 45 (91)	261 / 198 (459)	403 / 318 (721)

**Fig 1:**
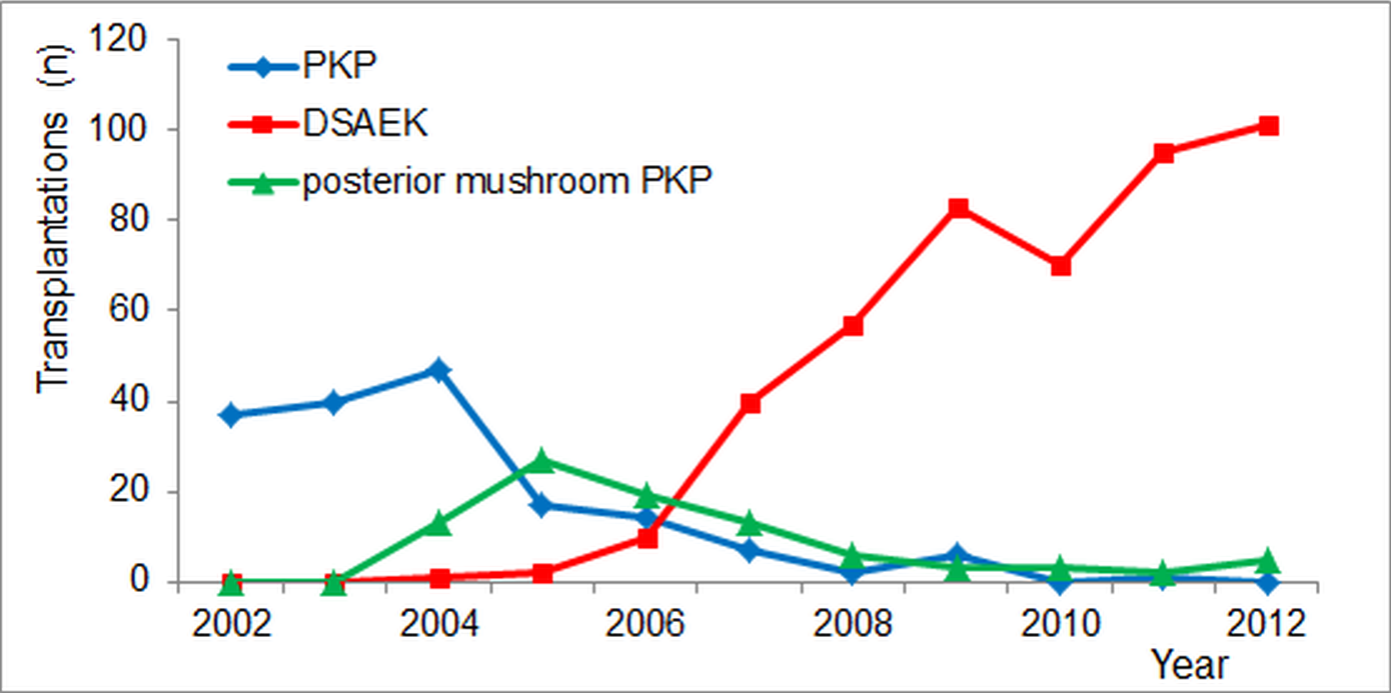
The annual number of cornea transplantations for Fuchs' endothelial dystrophy (N) in the Rotterdam Eye Hospital between 2002 and 2012. Penetrating Keratoplasty (PKP), posterior mushroom PKP, Descemet's Stripping Automated Endothelial Keratoplasty (DSAEK).

There were no statistical differences between the three groups with respect to gender (χ^2^-test, P = 0.58) or left or right eye operated (P = 0.54). Overall, subject age (mean ± SD) was 71.6 ± 9.1 years (range: 39.7–90.9) at surgery. Groups were not homogeneous with respect to age (*F* (2, 718) = 3.78, P = 0.02); there appeared to be a statistically significant difference between de posterior mushroom PKP group and the DSAEK group (73.6 ± 8.0 versus 71.0 ± 9.2 years; post-hoc Tukey, P = 0.035). The historical development with respect to technique preference is shown by the annual surgery frequencies in Fig 1. As can be read from this graph, PKP and inverted mushroom PKP as preferred surgical techniques performed for FED were gradually replaced by DSAEK during the period 148 from 2005 to 2007. No specific allocation rules were employed for the different surgical techniques, the gradual transition is merely a refection of not all surgeons changing simultaneously from penetrating to posterior lamellar transplantations.

### Graft survival

**Fig 2:**
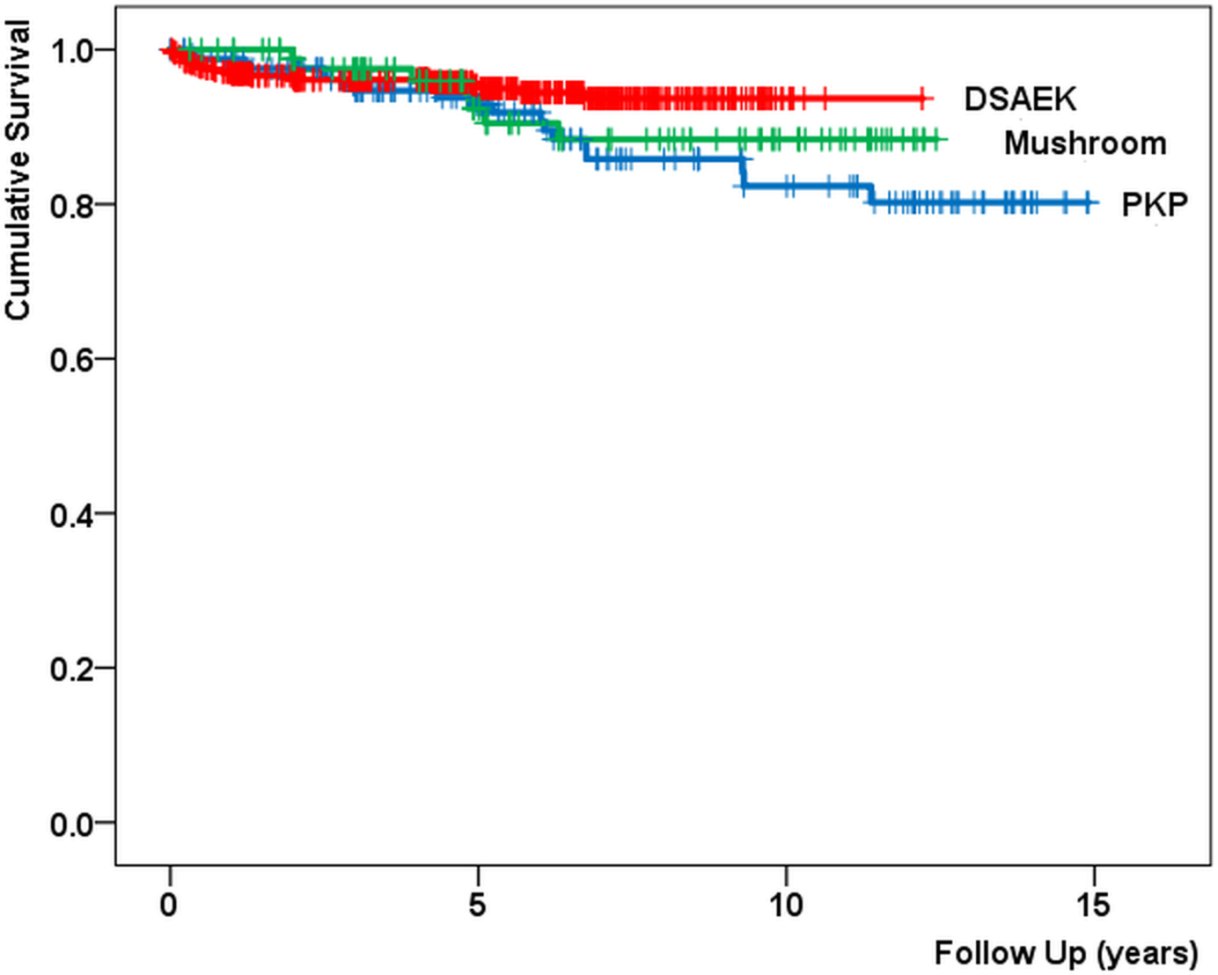
Graft survival for Penetrating Keratoplasty (PKP), posterior mushroom PKP (Mushroom) and Descemet Stripping Automated Endothelial Keratoplasty (DSAEK). Log-rank test: p = 0.12.

In total, graft failure occurred in 48 eyes: 19 (11%) in the PKP group, 7 (8%) in the posterior mushroom PKP group and 22 (5%) in the DSAEK group. The survival curves for the three techniques (Fig 2) did not show any statistically significant difference (log-rank test: P = 0.12). Mean follow-up period was 6.6 ± 4.3 years for PKP, 6.4 ± 3.6 years for posterior mushroom PKP and 4.9 ± 2.7 years for DSAEK. Primary graft failure occurred after 2 regular PKP and 11 DSAEK procedures (1.2 and 2.4%). Immunological rejection episodes occurred in 7 eyes (4.1%) after PKP, in1 eye (1.1%) after posterior mushroom PKP and in 4 eyes (0.9%) after DSAEK (P = 0.03). Potentially surgery-related adverse events that were reported (for respectively PKP, mushroom PKP and DSAEK) were bacterial or viral infection (7, 1, 1), ocular hypertension or secondary glaucoma (7, 1, 7), astigmatism correction (32, 12, 0). One posterior mushroom PKP eye had to be eviscerated.

### Visual acuity

Best spectacle-corrected visual acuity at baseline, and at 1, 2, 5 and 10 years for the three patient groups is provided in Table 2. Within all groups, visual acuity gain relative to baseline is highly significant. No significant difference between the visual outcome of PKP and posterior mushroom PKP was observed but the BSCVA after DSAEK procedures was significantly superior (at 2 years: *F* (2, 533) = 12.2, P << 0.001). Remarkably, however, the same was observed for preoperative vision (*F* (2, 712) = 16.4, P << 0.001). After 2 years, visual *improvement* relative to baseline, is not homogeneous between groups, i.e. 0.53 ± 0.61 logMAR (PKP), 0.55 ± 0.66 logMAR (posterior mushroom PKP) and 0.39 ± 0.40 logMAR (DSAEK; *F* (2, 532) = 5.5, P = 0.004) but subsets overlap.

**Table 2:** Best spectacle corrected visual acuity (BSCVA in logMAR, mean ± SD) differentiated by technique at baseline, and at 1, 2, 5 and 10 years postoperatively.

BSCVA	Preoperative	1 year	2 years	5 years	10 years
Penetrating Keratoplasty (PKP)	0.89 ± 0.53 (0.13) n = 168	0.42 ± 0.32 (0.38) n = 140	0.35 ± 0.29 (0.45) n = 121	0.43 ± 0.40 (0.37) n = 83	0.39 ± 0.31 (0.41) n = 32
Posterior Mushroom PKP	0.90 ± 0.58 (0.13) n = 89	0.42 ± 0.35 (0.38) n = 77	0.41 ± 0.42 (0.39) n = 70	0.40 ± 0.44 (0.40) n = 48	0.41 ± 0.28 (0.39) n = 13
Descemet Stripping Automated Endothelial Keratoplasty (DSAEK)	0.68 ± 0.41 (0.21) n = 458	0.31 ± 0.39 (0.49) n = 385	0.25 ± 0.26 (0.56) n = 346	0.24 ± 0.33 (0.58) n = 243	0.25 ± 0.19 (0.56) n = 13
Total number of eyes (proportion)	715 (99%)	602 (83%)	537 (74%)	374 (52%)	58 (8%)

**Fig 3:**
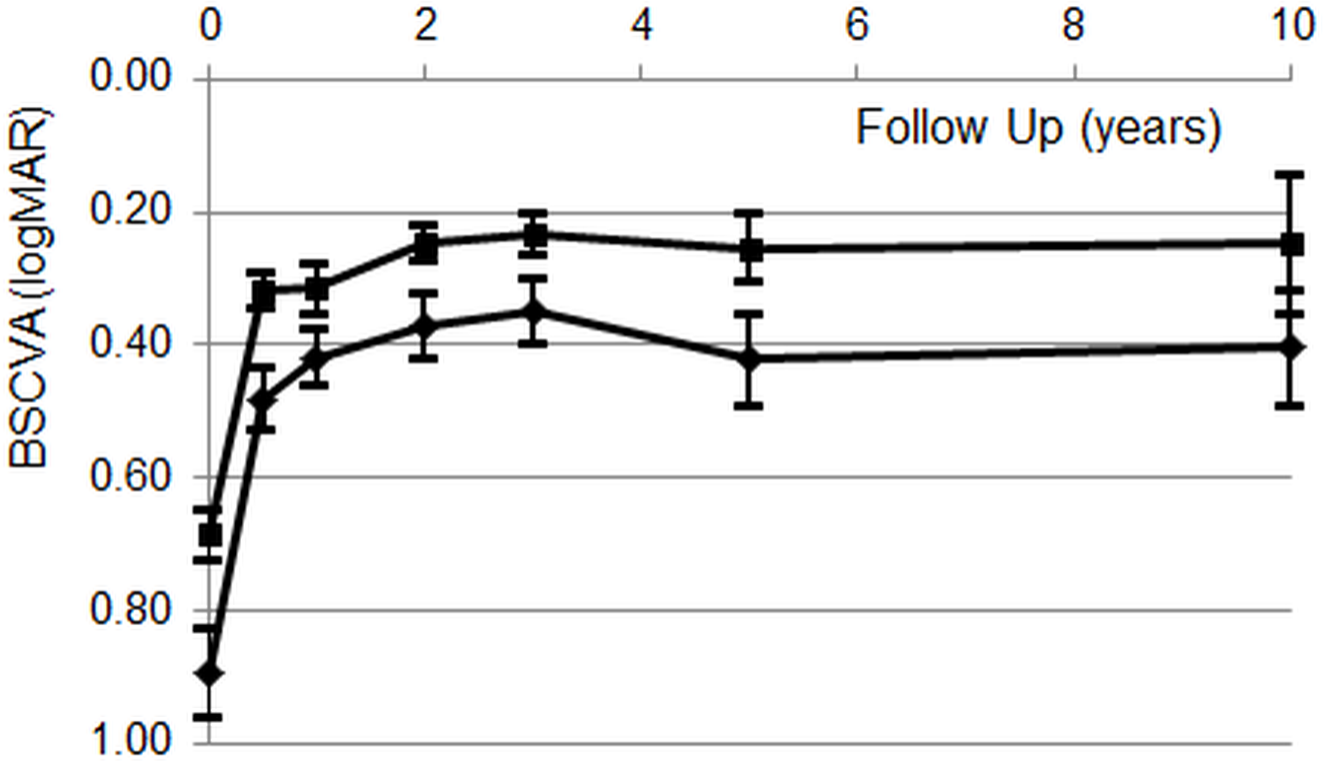
Average preoperative- and postoperative BSCVA after Descemet Stripping Automated Endothelial Keratoplasty (DSAEK; upper curve) and after pentrating keratoplasty (PKP & mushroom PKP; lower curve). Error bars represent 95% confidence intervals. As the BSCVA of PKP and posterior mushroom PKP were similar, both preoperatively and during the entire follow- up period, data of patients that underwent penetrating keratoplasty were combined.

In parentheses: mean logMAR visual acuity transformed to Snellen. Mean BSCVA of penetrating and posterior lamellar keratoplasty are shown in Fig 3.

### Surgical effect on refraction

Although the eyes that underwent PKP were somewhat more hyperopic (1.7 ± 2.8 D) than those that underwent posterior mushroom PKP (0.7 ± 2.2 D) or DSAEK (0.6 ± 2.3 D), the spherical equivalent of subjective refraction after the surgical intervention did not differ among the three techniques. At 2 years, these were 0.8 ± 3.6, 1.0 ± 3.6 and 1.1 ± 1.6 D respectively.

Astigmatism measurements are summarized in Table 3. Preoperatively, no significant differences were observed (Median test, P = 0.36). Postoperative astigmatism differed significantly among the three techniques (P << 0.001).

**Table 3:** Astigmatism (Diopters, mean ± SD) differentiated by technique at baseline and at 1, 2 and 5 years postoperatively.

	Preoperative	1 year	2 years	5 years
PKP	2.1 ± 2.4 n = 51	4.3 ± 3.0 n = 129	4.6 ± 2.7 n = 107	4.4 ± 2.7 n = 63
posterior mushroom PKP	1.3 ± 0.9 n = 31	4.5 ± 2.7 n = 69	4.5 ± 3.3 n = 55	4.4 ± 2.5 n = 37
DSAEK	1.5 ± 1.3 n = 323	1.7 ± 1.2 n = 317	1.7 ± 1.1 n = 226	1.8 ± 1.0 n = 176

**Fig 4:**
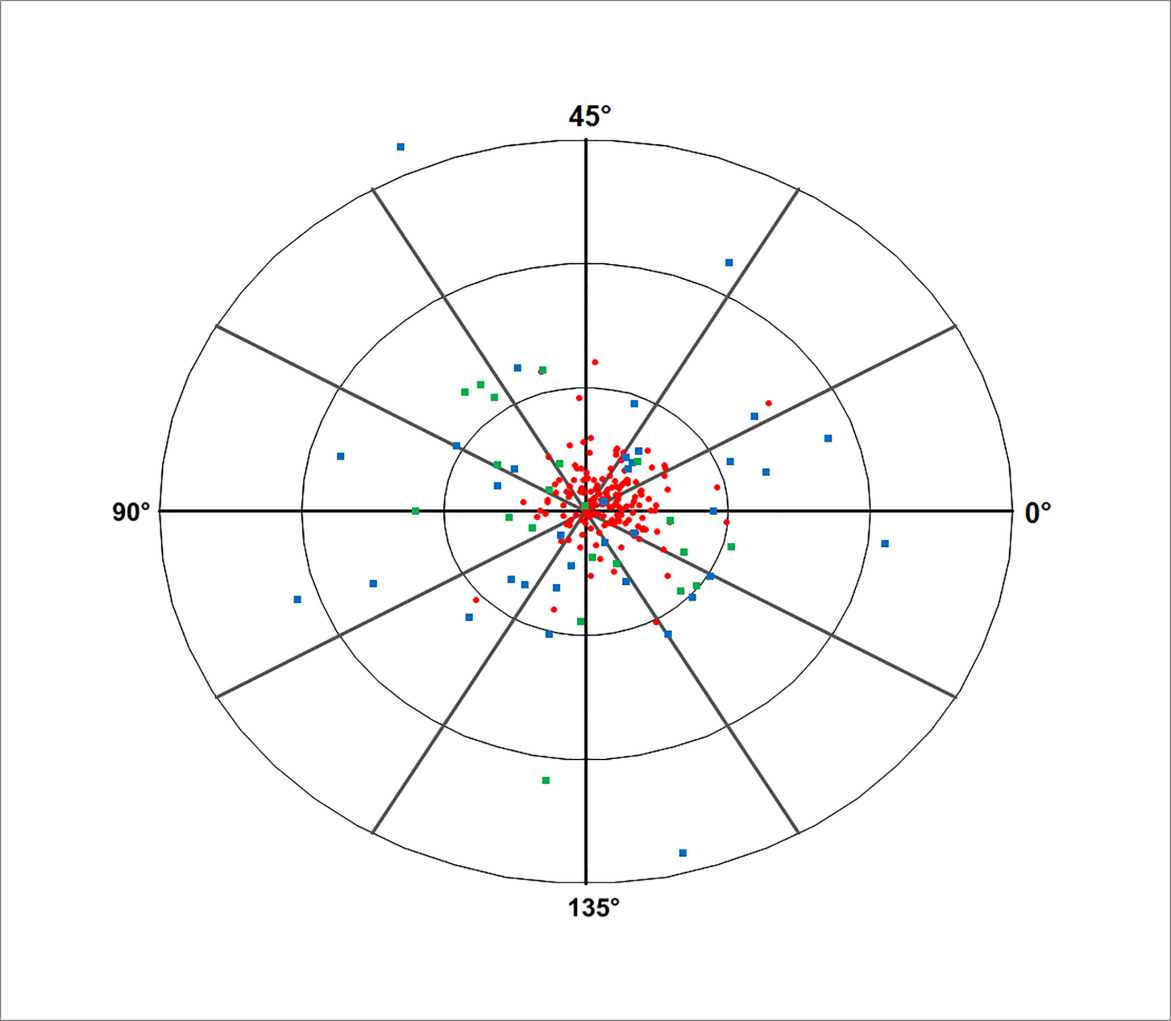
Double angle plot of surgically induced astigmatism showing the change of astigmatism at 2 years relative to baseline differentiated by the technique that was used: Penetrating Keratoplasty (PKP), posterior mushroom PKP, Descemet Stripping Automated Endothelial Keratoplasty (DSAEK). Rings at 5, 10 and 15 Diopters.

This substantial contrast with respect to surgically induced astigmatism between the two penetrating techniques on the one hand and DSAEK on the other is illustrated in Fig 4. For PKP (n = 35), the mean vector length of the surgically induced astigmatism was r = 5.54 D (with the centroid at r = 0.44 D and φ = 6˚). For mushroom PKP (n = 21), it was r = 4.12 D (centroid at r = 0.41 D and φ = 99˚). DSAEK (n = 167) induced much smaller changes of astigmatism: mean vector r = 1.68 D (centroid at r = 0.84 D and φ = 15˚).

## Discussion

In spite of the larger incidence of rejection episodes after penetrating grafts as compared to DSAEK, graft survival of the three corneal transplantation methods) did not differ significantly (Fig 2). The same conclusions could be drawn in other papers that described graft rejection and survival after PKP versus posterior lamellar grafts[10,11,12]. Concerning graft survival, similar results were reported for the Cornea Donor Study where PKP and DSAEK survival were analysed during the first 3 postoperative years[13], and for a 4 years’ follow up study of eyes with secondary endothelial failure[12]. Remarkably, cornea transplantation registry studies, with significantly larger numbers of eyes under investigation and a follow up of 2–3 years, indicate inferior results after DSAEK as compared to PKP[14,15]. The same registry studies give contradictory conclusions concerning the role of the surgeon's experience on graft failures after DSAEK [14,15]. Although the DSAEK surgeries in our study included the learning curves of all four surgeons, the incidence of primary graft failure in our DSAEK cohort (2.4%) corresponds with previously published rates ranging from 0–6% [16,17].

Although posterior lamellar techniques have been accepted as a useful surgical option in endothelial disorders[18], only few comparative studies including pre- and postoperative BCVA measurements were published. A small retrospective study which included 12 patients with PKP performed on one eye and DSAEK on the contralateral eye reported better BSCVA after DSAEK surgery. As in our study, pre-operative BSCVA was significantly better in the DSAEK group[19]. The same holds true for a nonrandomised prospective study by the same authors including 48 PKP- and 45 DSAEK procedures[16]. Another study comparing 20 eyes undergoing DSAEK with a historical group of 20 eyes that underwent PKP however reported a comparable preoperative BSCVA, in combination with a significantly better BSCVA after DSAEK[20].

Two large national registry studies (UK & Australia) were not able to formulate concordant conclusions on the superiority of either lamellar or penetrating techniques with respect to the combined outcomes of BSCVA and graft survival [14,15]. Factors such as surgical experience may have played a role[14] as techniques for posterior lamellar grafting for endothelial disorders have only recently become available and learning curves have to be taken into consideration. Additionally, posterior lamellar grafting techniques are still in development. For example, in our center the surgical technique for DSAEK as it was performed in this study is already replaced by ultrathin DSAEK with the donor tissue prepared by the eye bank, and also DMEK has become available. For these latter techniques mid- and long-term results as described in the current study will only become available after several years from now.

As expected, this comparative studies on two penetrating- and one posterior lamellar surgical method (DSAEK) for FED demonstrated a significant effect of all three techniques on visual rehabilitation. As nylon sutures were all removed from PKP eyes in our clinic within 24 months, large changes in BSCVA after 2 years did not occur (Table 2). Compared to the two penetrating techniques, both pre- and postoperative BSCVA were significantly better in the DSAEK group (Fig 3). Analysis of the *improvement* in BSCVA (pre-operative versus 2 years postoperatively), although statistically inconclusive, appears to indicate that the visual gain after DSAEK is not superior to that after penetrating keratoplasty. Two recent studies reported similar results with respect to preoperative BSCVA and vision recovery after penetrating keratoplasty and DSAEK respectively [21], [8]. As in our patients, pre-operative and postoperative BSCVA were significantly better in the DSAEK group.

This suggests that candidates for DSAEK in our study, as well as in the other two studies, were selected at an earlier stage of FED than for penetrating keratoplasty. It has been pointed out that this effect should be taken into consideration when analysing BSCVA outcomes[21], [8].

This notion of surgical intervention at an earlier stage of FED may also explain the small but significant difference in age we found between (mushroom) PKP and DSAEK: 72.8 ± 9.0 versus 71.0 ± 9.2; P = 0.01. Although this may appear to be only a small difference it occurs in an elderly population with a relatively high incidence of other ocular disorders such as macular degeneration and postoperative cystoid macula oedema, disorders which may have a significant effect on BSCVA. Currently the tendency to operate eyes with FED at an earlier stage is generally acknowledged and appears to be justified because DSAEK (or any other posterior lamellar technique) is relatively safe. Additionally, visual recovery is faster and refractive results after DSAEK are superior to those after penetrating corneal transplants.

The penetrating techniques, PKP and posterior mushroom PKP, showed significantly larger vectors of surgically induced astigmatism than DSAEK (Fig 4). While the vectors of both penetrating techniques appear to be randomly distributed in all directions, the DSAEK vectors tend to point to the right which corresponds to a surgically induced change in astigmatism in the direction of the vertical axis. This can be explained by the fact that for the DSAEK technique a tunnel close to 90˚ was applied in the large majority of cases. The low change in astigmatism after DSAEK as compared to the penetrating corneal transplantation techniques (Fig 4, Table 3.) is in concordance with other studies[16,20,22,23]. As astigmatism after PKP and inverted mushroom PKP (Fig 4, Table 3) were very similar, it may be questioned whether inverted mushroom PKP is superior to conventional PKP in terms of (induced) astigmatism and visual function.

In conclusion, there was no significant difference in graft survival between the three techniques; after DSAEK, BSCVA was significantly better while surgically induced astigmatism was significantly lower. In addition, posterior lamellar grafting has the advantage of a relatively fast recovery and a much safer wound configuration.

As compared with penetrating corneal transplantation techniques, the better BSCVA results after DSAEK are probably explained by patient selection at a slightly younger age and concurrently at an earlier stage of the FED disease process. However, because this BSCVA improvement persists for a considerable period of time, it may substantially contribute to the patient's quality of life. Together with the other benefits mentioned above, and provided that enough donor tissue is available, the earlier intervention appears to be justified indeed. For a relatively numerous category of patients comprising potential candidates for keratoplasty this may lead to a significant increase in ‘quality years’, particularly if the results of future comparative studies of recent developments of posterior lamellar keratoplasty, such as ultrathin DSAEK and DMEK, corroborate the expectation of further improvement.

## Supporting information

S1 File PKPvsMushroomvsDSAEK_Plos_dataset.xlsx. Supporting information on raw follow-up data concerning demographics, graft survival, BSCVA, and refraction (sphere, cylinder, axis). Gender: 1 = male, 2 = female; Surgery: 1 = PKP, 2 = DSAEK, 3 = inverted mushroom PKP; Cornealgraftlastfu: 7 = failure; LM = LogMAR; K1 = keratometry in dioptres on flattest meridian; K2 = keratometry in dioptres on steepest meridian.
(XLSX)
